# Artificial neural networks enabled by nanophotonics

**DOI:** 10.1038/s41377-019-0151-0

**Published:** 2019-05-08

**Authors:** Qiming Zhang, Haoyi Yu, Martina Barbiero, Baokai Wang, Min Gu

**Affiliations:** Laboratory of Artificial-Intelligence Nanophotonics, School of Science, RMIT University, Melbourne, VIC 3001 Australia

**Keywords:** Laser material processing, Integrated optics

## Abstract

The growing demands of brain science and artificial intelligence create an urgent need for the development of artificial neural networks (ANNs) that can mimic the structural, functional and biological features of human neural networks. Nanophotonics, which is the study of the behaviour of light and the light–matter interaction at the nanometre scale, has unveiled new phenomena and led to new applications beyond the diffraction limit of light. These emerging nanophotonic devices have enabled scientists to develop paradigm shifts of research into ANNs. In the present review, we summarise the recent progress in nanophotonics for emulating the structural, functional and biological features of ANNs, directly or indirectly.

## Artificial neural networks: tapping in to the power of light

Technologies that manipulate light at the nanoscale will help researchers develop artificial neural networks (ANNs) with uses including brain disease research and machine learning. Despite advances in neuroscience, understanding the human brain remains a considerable challenge. Constructing physical or computer-based ANNs can help scientists analyse brain function and harness its power. Min Gu and colleagues at RMIT University in Melbourne, Australia, reviewed research into emerging ANNs enabled by nanophtonics that harness photons’ ability to carry vast amounts of information. Three-dimensional printing and laser writing techniques are allowing researchers to fabricate tiny optical or electronic components for building artificial neurons and ANN scaffolding platforms. Another all-optical ANN design uses multiple layers of diffractive holograms to create a highly efficient machine learning engine. Transplantable ANNs combining nanophotonic technology such as nanosensing with biological tissues could one day help study and treat severe brain disorders or injuries.

## Introduction

The human brain is the most complex biological organ in the universe and remains unknown to us. Several countries have started large programmes or alliances on brain science^1^. Together, these initiative projects will undoubtedly lead to major breakthroughs in understanding how the human brain functions, which will provide possible solutions to curing brain-related diseases. These projects will also inspire neuromorphic computing to meet growing demand for artificial intelligence^2^ to build a machine that mimics the capability of humans towards various applications.

To obtain a better understanding of the brain, investigations of biological neural networks (BNNs) have been widely carried out to study the biological, structural and functional features of the brain. One category of this research involves the use of advanced electrophysiology and imaging techniques to map and study the activities of BNNs, which include: microelectrodes^3^, electroencephalograms (EEGs)^4^, magnetic resonance imaging^5^, computed tomography^6^, electron beam microscopy^6^ and super-resolution fluorescence microscopy^7^. This category allows an understanding of the operation of the brain via a bottom–up approach using the large amount of data obtained through BNN studies. Another category of BNN research focuses on the building of artificial neural networks (ANNs) that could emulate the biological, structural and functional features of BNNs. This category uses simplified and controllable models to test new theories of brain functions derived from the data of BNNs and to test new drugs on the brain-related diseases. ANNs can also provide brain-like computing platforms for artificial intelligence with higher efficiencies.

Building ANNs includes research in the fields of software simulations based on conventional von Neumann computers^8,9^ and hardware simulations, such as the implementation of ANNs indirectly based on electronics^10,11^ and photonics^12–15^ and the direct growth of ANNs with biological neuron cells^16^. Although versatile supercomputers based on conventional von Neumann computers are now available to conduct millions of operations, the software simulation of ANNs with von Neumann computers in 100% real time and at the scale of the whole human brain has not yet been achieved and would consume at least 500 MW of energy^17^, not to mention its huge size. These drawbacks are mainly owing to the serial nature of von Neumann computers, which is fundamentally different from how the BNNs work. The development of ANNs based on hardware is an important step for the practical realisation of ANNs.

**Fig. 1:**
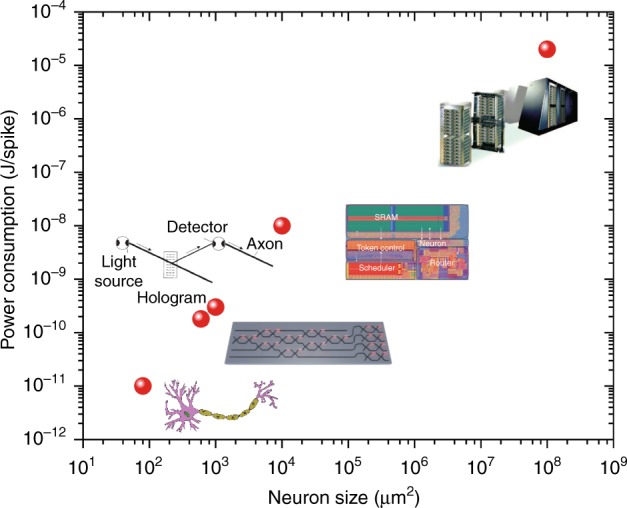
Development of artificial neural networks^8,10,12,15^

Nanophotonics—the study of the behaviour of light and the light–matter interaction at the nanometre scale—is of great importance to demonstrate hardware-based ANNs. In fact, the interplay between nanophotonics and ANNs has already generated new fields. The application of software-based ANNs in nanophotonics has enabled new realms in automatic optical sensing^18^, automatic optical microscopy imaging^19^ and the inverse design of photonic devices^20^. Nanophotonics is a highly promising tool for studying BNNs with optical imaging and optogenetics. The development of optical imaging—especially the invention of far-field super-resolution optical microscopy (awarded the 2014 Nobel Prize in Chemistry), has initiated research into imaging neural networks at the nanoscale^21,22^. Based on the same principle as far-field super-resolution imaging, a two-beam optical nanolithography technique, termed super-resolution photoinduction-inhibited nanolithography (SPIN), has been developed to fabricate features as small as 9 nm^23^, which reaches the feature size of BNNs. Such a technique has the potential to develop the three-dimensional (3D) direct nanoprinting of ANNs with high complexity and capacity. In addition, the photon is an important information carrier for ANNs, exhibiting a broad bandwidth and low transmission scattering compared with the electron. The development of ANNs based on nanophotonic devices has opened a new avenue to achieve orders-of-magnitude improvements in both computational speed and energy consumption over existing solutions based on electronics (Fig. 1).

In the present review, we discuss the recent advancements in nanophotonic techniques for the development of ANNs that mimic and study the structural, functional and biological features of BNNs. First, we describe the nanophotonics-enabled indirect ANNs based on electronics and photonics, including the laser writing of electronic devices for ANNs and the development of nanophotonic devices for ANNs. After that, we briefly summarise the recent advancement in building ANNs based on biological neurons with controlled topology. Finally, we summarise the nanophotonic techniques used for the imaging and signal detection of ANNs based on biological neurons.

## Indirect ANNs enabled by nanophotonics

The construction of intelligent machines that mimic BNNs has been pursued since soon after the invention of the modern computer. Most of the research into ANNs is based on software simulation using von Neumann computers. The concept of mimicking BNNs with electronic or photonic hardware, which is also called neuromorphic computing, was introduced in late 1980s^12,24^. Compared with the ANNs directly based on biological neural cells, these ANNs rely on electronic or photonic systems containing artificial neurons to indirectly mimic the neurobiological architectures presented in BNNs.

### Working principles of indirect ANNs

**Fig. 2:**
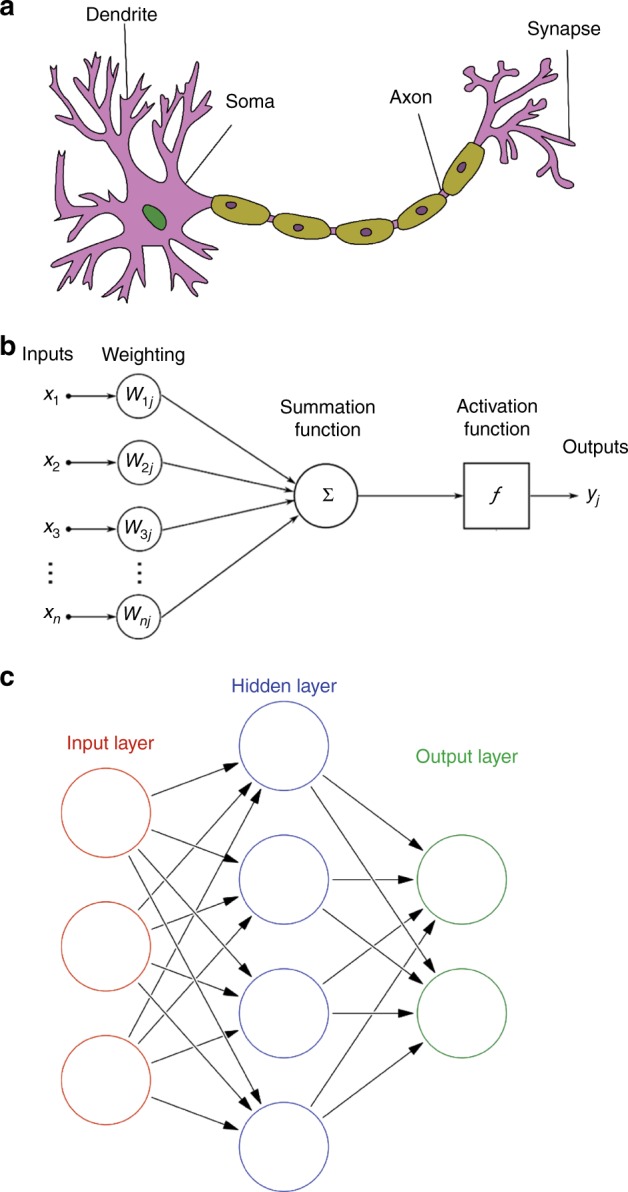
Working principle of artificial neural networks. **a** A schematic drawing of biological neurons^25^. **b** A schematic drawing of artificial neurons^26^. **c** The structure of feed forward artificial neural networks^27^

The biological counterparts of artificial neurons are biological neurons, which represent the building blocks of BNNs. The BNNs consist of billions of neurons of different types and sizes. Figure 2a shows a schematic of a simplified biological neuron^25^ with the four basic fundamental units—dendrites, axons, somas and synapses.

ANNs follow a simplified model inspired by BNNs. The unit of this model, a simple formalised artificial neuron introduced by McCulloch and Pitts^26^, acts as a computational element in the ANN. The execution of a task involves the parallel activation of a large number of artificial neurons. As shown in Fig. 2b, one or more inputs (*x*) from the other neurons were sent into an artificial neuron and the inputs are summed up to produce outputs (*y*) to the other neurons on axons. Separated by weighted (*w*), a non-linear function known as an activation function or transfer function (*f*); the activation function can be a step function, Sigmoid function, etc. The mathematical form of the artificial neuron is as follows:

1 $$y_j = f\left( {\mathop {\sum}\nolimits_{i = 0}^n {w_{ij}x_i} } \right)$$

The functions of the key components (axon, dendrite, soma and synapse) in ANNs are summarised in Table 1. The analogy between artificial neurons and biological neurons holds that the interconnections of the signals stand for the axons and dendrites, the summation and threshold approximate the activation in the soma, and the connection weights and memory represent the synapses.

**Table 1:** Summary of the key components of artificial neural networks

Components	Functions	Photonic devices
Axon and dendrite	Interconnection	Free space^12^, waveguides^13–15^
Soma	Summation and thresholding	Photodetectors^12–15^, electric-optic modulators^14^, light sources (LED and laser)^12^, optical amplifiers^52^, saturable absorbers^52^, optical bistable devices^53^
Synapse	Weighting	Hologram^12^, MZI^15^, micro-ring resonators^14^
	Memory	Electronic memories^12–15^, reversible optical memories^54^

Artificial neurons can be organised in any topological architecture to form ANNs. A common architecture, the feed forward ANN^27^, is shown in Fig. 2c. In this network, the information moves only in the forward direction. A series of artificial neurons are integrated to form the layer of input. Within the ANNs, the input signal is passed through one or more hidden layers. At the end of this structure, the output layer provides the results. Different from the current von Neumann computer that performs tasks by the pre-design of the programme, the ANN can learn to run a task through a sequence of training with examples. For example, one of the typical learning processes is the supervised learning method by updating weights with the backpropagation errors between known target values and the output values^28^.

The most common unsupervised learning method is the spike timing-dependent plasticity (STDP) algorithm^29^, inspired from the spiking nature of BNNs. Recent neurological research has shown that biological neurons encode information in the timing of single spikes^29^. Functionally, STDP constitutes a mechanism for implementing a Hebbian learning rule^30^, allowing for the learning process without supervision.

### Laser-written electronic memristors for ANNs based on electronics

The ANNs based on electronics were first demonstrated with very large-scale integration (VLSI) systems^24^. The electronic wires on the chips can be used as electronic axons and dendrites. The combination of several transistors in VLSI are used to approximate electronic synapses and somas. ANNs with a large amount of artificial neurons based on VLSI have been developed^11^. However, the integrated level of such a kind of ANN is still low owing to the complexity of the architecture. Memristors, the fourth basic element in electronic circuits predicted in 1971^31^ and demonstrated in 2008^32^, provide a new path to demonstrate ANNs with a high integration of electronic devices.

**Fig. 3:**
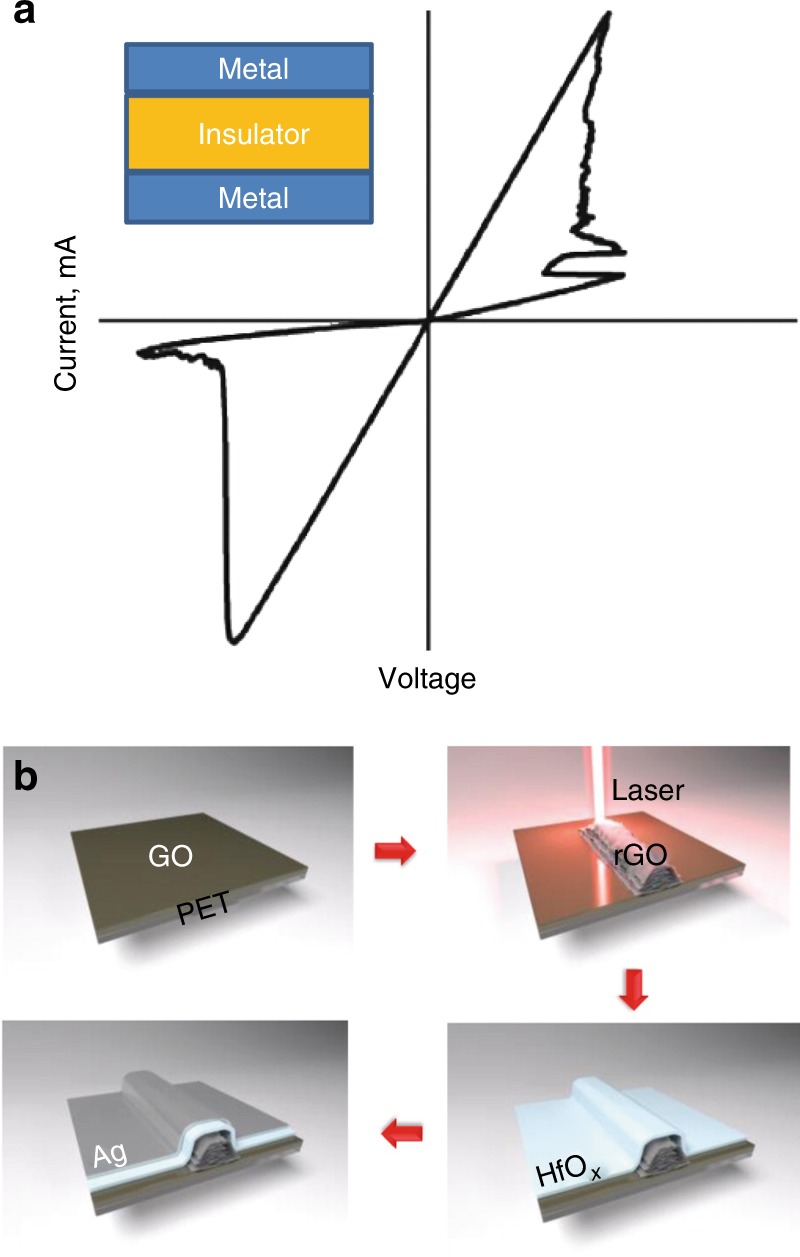
Introduction of memristors. **a** Typical switching behaviour of a memristor^32,33^. Inset: a memristor formed with a metal/insulator/metal structure. **b** A schematic drawing of the direct laser writing of a memristor on a PET substrate^42^

Memristors are passive two-terminal circuit elements, usually with a metal/insulator/metal structure^33^ (Fig. 3a). Mimicking a biological synapse, the resistance of a memristor can be adjusted by tuning input amplitude of the charge or flux. It has been demonstrated that the synaptic functions can be implemented experimentally with nanoscale silicon-based memristors^34^. The learning function has been demonstrated in a circuit composed of memristor synapses and complementary metal-oxide semiconductor neurons^35^. An artificial soma function has been demonstrated with a neuristor built using two nanoscale Mott memristors^36^. An ANN unsupervised learning for pattern classification based on memristors has recently been demonstrated recently^37^.

Nanophotonics offers a new solution to fabricate memristors by the laser reduction of graphene oxide. Graphene has received significant attention owing to its superior electronic and optical properties^38^. Direct laser writing (DLW) with laser reduction can realise the simultaneous direct growth and patterning of rGO. Various rGO electronic devices such as capacitors^39^, gas sensors^40^ and multifunctional devices^41^ have been demonstrated.

A memristor based on rGO has been experimentally demonstrated. DLW is used to realise rGO fabrication for use as the bottom electrode of the memristor on flexible substrates (Fig. 3b). An insulating layer of 10 nm HfOx is blanket deposited by thermal evaporation. The top electrode is made by curable silver paste or using thermal evaporation. The fabricated Ag/HfOx/rGO structure exhibits stable switching up to 100 cycles^42^. Metal-free memristors have been fabricated through the DLW process with rGO/g-C3N4-NSs/rGO thin films^43^.

### Introduction of ANNs based on photonics

Photons are an ideal information carrier because of their specific properties, such as inherently massive parallelism, fast propagation speed and no side effects of mutual interference. Optical signals can be multiplexed in time^44^, space^45^, polarisation^45,46^, angular momentum^47^ and wavelength^45,48^ domains, and optical technologies may overcome the problems inherent to electronics. Thus, the research and development into optical interconnect technology has already led to the replacement of copper connections in computer chips and data centres by optical waveguides or fibres^49^, which can potentially improve the performance of ANNs based on software simulation and electronics. An ANN based on reservoir computing^50^ has been demonstrated with temporal multiplexing in a single optical fibre. ANNs based on nanophotonics offer a promising alternative approach with a faster response and lower power consumption.

ANNs based on nanophotonics can be achieved by providing key optoelectronic or optical components for the functions of ANNs (Table 1). Nanophotonic technology has several advantages in making interconnections with free space and waveguides, specifically with regard to broad bandwidth, low-loss and low-crosstalk. Photonic somas can be achieved either by optical–electrical and electrical–optical signal transferring with photodetectors^12,14,15^, electro-optic modulators^14^ and light sources (LED and laser)^51^ or by an all-optical process with lasers^51^, optical amplifiers^52^, saturable absorbers^52^ and optical bistable devices^53^. The weighting function can be achieved by optical switches such as holograms^12^, Mach–Zehnder interferometers (MZIs)^15^ and MRRs^14^. The memory function to record the weights in a synapse can be achieved by electronic memories or non-volatile optical memories^12,54^.

### ANNs based on holography in free space

**Fig. 4:**
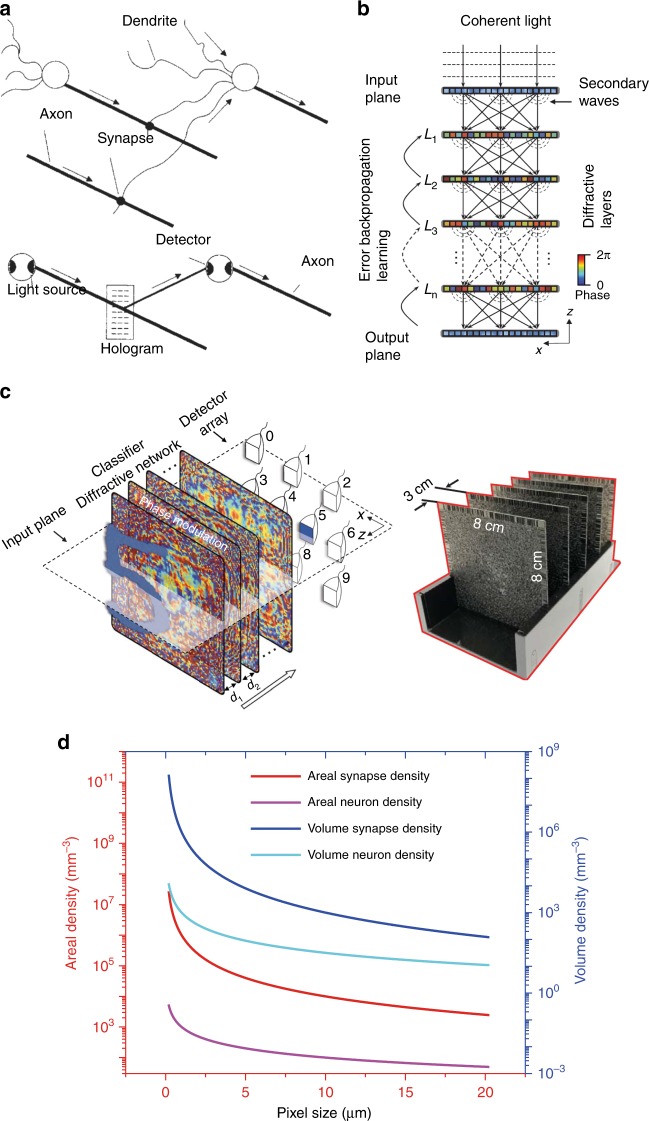
Architectures of artificial neural networks based on holography. **a** An ANN based on holography and composed of optoelectronic neurons^12^. **b** An ANN based on holography and composed of multiple passive layers^56^. **c** An ANN based on holography and composed with multiple passive layers enabled by 3D printing^56^. **d** Areal and volume densities of the artificial synapses and neurons of ANNs based on holography with different pixel sizes

In ANNs based on holography, the full parallelism of light in free space can be used and additional functionalities become available. These strengths have been recognised for many years, and early implementations utilised reconfigurable holograms for forming interconnections between optoelectronic artificial neurons^12^. The architect of the ANNs based on holography with optoelectronic artificial neurons is shown in Fig. 4a. The light sources and photodetectors combine as optoelectronic neurons. The input signal is generated by the modulation of the light source. The holographic interconnection is achieved by optical diffraction with holographic gratings. Holography gratings can be produced by using spatial light modulators (SLMs)^55^ and the photorefractive effect^12^. The detectors integrate the optical signal and transfer it into an electronic signal. The threshold function is achieved by processing the electronic signal with electronic circuits. Although free-space optics provide the media for the highly integrated large number of free-space interconnections and data communication at the speed of light, the hologram provides an approach for storing and implementing the weight matrix data between fully connected neural layers.

Recently, a all-optical deep learning framework in the terahertz region (0.4 THz) has been demonstrated as an all-optical deep learning network, in which the ANN consists of multiple layers of a hologram fabricated by 3D additive printing^56^. The ANN is created by using several layers of a diffractive surface, where each point on a given layer represents an artificial neuron (Fig. 4b). The learning process of such an ANN is based on a computer simulation to design the phase modulation of each layer (Fig. 4c), which can be fabricated with a 3D printer and based on plastic materials. The ANN cannot be tuned after the fabrication process. Compared with previous ANNs based on holography composed of optoelectronic neurons, this kind of ANN is based on all passive elements.

The ANNs based on holography can be easily scaled up using various fabrication techniques including electron beam lithography and photolithography. ANNs based on holography provide the potential for a large number of artificial synapses and neurons owing to the high parallelism in free space. If we assume that the artificial neurons in two adjacent layers of an ANN are all arbitrary interconnected. The relationship between the density of artificial neurons (*Dn*) and the density of artificial synapse (*Ds*) is:

2 $$D_n = \root {2} \of {{D_s}}$$

The maximum number of artificial synapse via holography is equal to the maximum number of gratings that can be supported by the hologram^12^. From the sampling theorem, the number of sinusoids or gratings that can be recorded in the medium is equal to the number of samples. Considering the pixel size of a hologram is *δ* in all dimension (*δ* *>* *λ*), the maximum number of independent artificial synapse is *V/δ*^3^ in a volumetric hologram or *A/δ*^2^ in a thin hologram, where *V* and *A* are the volume and area of the hologram, respectively. As shown in Fig. 4d, for a commercial SLM with a pixel size of 4 µm, the areal density of synapses (*D*_*sa*_) is 6.25 × 10^4^/mm^2^. A recent advancement in holography induced by laser has demonstrated a hologram with a pixel size of 0.55 µm^46^, which corresponds to a synapse density of 3.31 × 10^6^/mm^2^. As this hologram is induced by the tight focusing of laser light, it has potential to achieve a volume synapse density (*D*_*sv*_) of 6.01 × 10^9^/mm^3^ if a volume hologram can be induced by laser in three dimensions. The corresponding maximum areal neuron density (*D*_*na*_) and volume neuron density (*D*_*na*_) can be calculated by Eq. (2), as shown in Fig. 4d. A recent advancement in the SPIN technique, with a feature size of 9 nm^23^ and optical signal multiplexing^45,47^, can further improve the density of synapses and neurons.

Apart from plastic materials and SLMs, ANNs based on holography can be achieved with different materials (Table 1). The 3D two-photon polymerisation of polymers^23^ and chalcogenide glass^57^ can be used for ANNs based on holography. Holography imaging has been demonstrated by DLW with the photoreduction of graphene oxide^46^. A hologram with a thickness of 60 nm using a topological insulator material has been demonstrated by DLW^58^. ANNs based on the materials above are write-once only owing to the non-reversible photoinduced effect, requiring learning with pre-design structures in computers.

Reversible holograms have been demonstrated with photorefractive polymers^59^ and photorefractive polymers synthetised with nanoparticles^60^. A hologram based on an ultrathin layer of phase change material Ge_2_Sb_2_Te_5_ (GST)^61^ is also demonstrated. These holograms can be erased and written by the DLW technique owing to the thermally reconfigurability of GST. These reversible holography effects open a new avenue for ANNs with a closed-loop learning capability, which allows direct updates of the weighting in ANNs after receiving feedback from the outputs.

### ANNs based on photonic circuits on a chip

Integrated photonic circuits on a chip are an ideal platform for ANNs with a high compactness and high stability. As the photonic axons and dendrites, the low-loss waveguides transmit the optical signal. The development of integrated lasers, photodetectors and optical non-linear devices can serve as photonic somas. The weights can be achieved in the optical domain by using reconfigurable optical switches or splitters, which utilise the temperature dependence of the refractive index (the thermo-optics effect) to realise reconfigurable functionality by heating the devices in most of current devices.

**Fig. 5:**
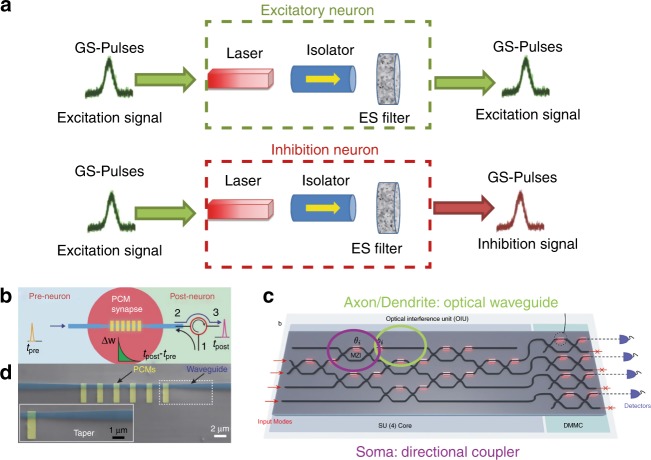
Artificial neural networks based on photonic circuits on a chip. **a** Schematic representation of the basic operation of an excitatory and inhibition neuron with an integrated semiconductor laser on a photonic circuit^51^. **b** Schematic drawing of artificial synapses^62^. **c** The architecture of an artificial neural network based on photonic circuits^15^. **d** Integrated phase change memories on a photonic circuit as artificial synapses^62^

Interest in integrated lasers with neuron-like-spiking behaviour has flourished over the past several years^51^. Biological neurons use spikes to send information. Recently, neurological research has discovered that information in biological neurons are encoded in the temporal domain of single spikes^29^. Lasers operating in the excitable regime are perfectly dynamic candidates but are ~ 8 orders-of magnitude faster. However, most experimental work has focused on isolated neurons or single chain of neurons (Fig. 5a).

Isolated artificial synapses on photonic circuits have been demonstrated by integrating a phase change memory (PCM) on an optical waveguide^62^ (Fig. 5b). As shown in Fig. 5b, the synapse is based on a tapered waveguide with PCM regions on top. The synaptic weight can be controlled by changing the number of optical pulses sent through the waveguide.

In parallel with the work on isolated artificial neurons and synapses, recent research has also investigated the capability of photonic circuits to mimic neural networks, focusing on the networks with connectivity to multiple neurons and layers. An ANN scheme based on silicon waveguide interconnections has been demonstrated by using a reconfigurable MZI to control the weights between the interconnections of neurons (Fig. 5c), which are laser and photodetectors^15^. Recently, a “broadcast-and-weight” scheme has been demonstrated to use wavelength-division multiplexing (WDM) to support a large number of reconfigurable interconnections enabled by micro-ring resonators (MRRs) on a silicon photonic chip^14^. A 294-fold acceleration against a conventional von Neumann computer is predicted.

The size of the reconfigurable MRRs or MZIs is from 625 to 20,000 µm^2^, resulting in a synapse density of 50–1600/mm^2^. The neuron density is at the same level and is based on the size of the integrated lasers and photodetectors on the chip. As mentioned in the previous paragraphs, current ANNs utilise the thermo-optics effect in reconfigurable MZIs or MRRs to realise artificial synapse functionality by heating the devices, resulting in an additional power consumption of 10 mW per synapse to maintain the reconfigurable MRRs or MZIs. However, the weights can be set by integrated on-chip non-volatile memory, such as a PCM, which requires no power to maintain.

### Future perspectives

The ANNs based on photonics have a significantly higher rate and lower computation energy compared to those of their electronic counter parts. ANNs based on holography can achieve an ultrahigh density of synapses owing to the high parallel processing in free space. Nonetheless, current ANNs based on photonic circuits cannot compete with the density of electronics. However, novel implementations of ANNs based on nanoscale optical data storage^63^, photonic crystal nanocavity^64^ and plasmonic nanocavity^65^ technologies may become important to the future scaling down of the artificial neuron and synapses on a chip to the diffraction limit scale (~ 1 µm) or even beyond the diffraction limit. Three-dimensional photonic integration enabled by DLW could enable ANNs with larger number of artificial neurons and synapses by adding another spatial degree of freedom. Furthermore, the feeding of input signals through temporal multiplexing would be able to realise ANNs with larger number of artificial neurons and synapses effectively.

Apart from the demonstration of single lasers or laser chains, the coding of ANNs based on photonics has been demonstrated using analogue optical signals. ANNs based on photonics can be coded with the spiking method to further improve the signal robustness and enable unsupervised learning. Current ANNs based on photonics are derived from simplified artificial neuron models. The performance of ANNs can be further improved with a better understanding of the working mechanism of BNNs.

## Direct ANNs enabled by nanophotonics

One of the fundamental interests in building ANNs lies in the possibility of unravelling the myth in the interactions between neurons. Building well-defined ANNs based on biological neurons with controlled topology (micro-platforms)^66^ can be used for studying neural activities such as axonal path finding and synaptogenesis, drug screening and targeting, and recreation computational units based on living cells^67^. Furthermore, transplantable ANNs^68^ consisting of biological neural tissue might offer the capability to treat several diseases that interrupt the connectome in neural systems, including Parkinson’s disease, traumatic brain injury, stroke and brain tumour excision^69^. A crucial unit for the successful building of ANNs based on intricate 3D bio-tissue is the manufacturing and adoption of well-designed 3D scaffolds. Successful scaffolds in tissue engineering applications rely critically on the physical and chemical characteristics of microstructures. Nanophotonics is the key enabling technology for the building of 3D scaffolds. In this chapter, we summarise different approaches and materials that are available to build ANNs enabled by nanophotonics.

### Fabrication of direct ANNs in two dimensions

**Fig. 6:**
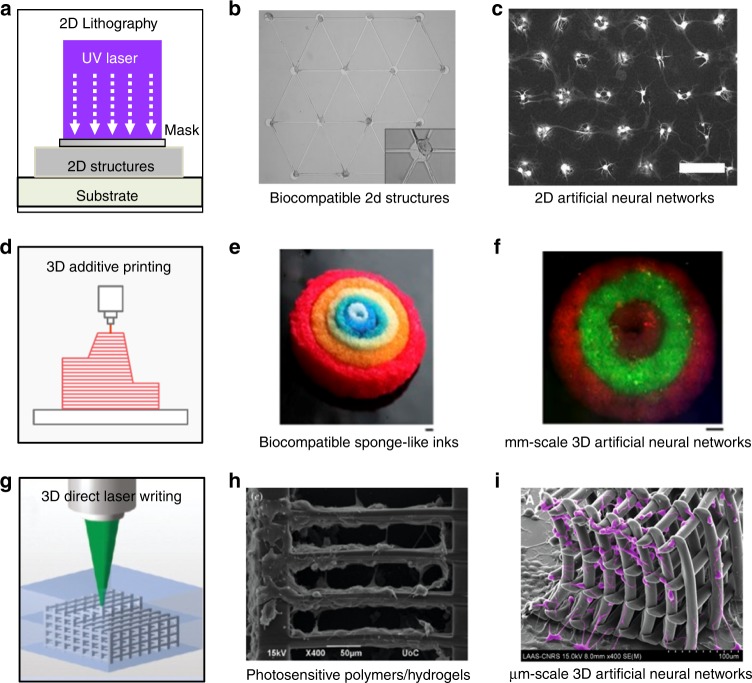
Recent developments in building artificial neuronal networks based on biological neurons with controlled topologies at different scales using different techniques **a–c** Steps in the creation of 2D ANNs using 2D fabrication technologies^72^ (photolithography, electron beam lithography, and micro-imprinting). **d–f** Millimetre-scale 3D ANNs created by 3D additive printing^79^. **g–i** The creation of micrometre scale 3D ANNs using 3D direct laser writing^87^

The mechanical and geometrical features of the surrounding structural matrix can have an impact on the structure and functions of the neurons^70^. To create well-defined direct ANNs with a desired topology, micro-platforms that emulate the structural features of BNNs should be created on a biologically compatible substrate, as shown in Fig. 6a–c. Different lithographic techniques have been utilised to create 2D microstructures on biologically compatible materials.

Soft lithography can be used to build small hutches to trap neurons on the surface of electrodes^71^. Similar structures such as troughs and wells can be etched into a silicon wafer. A triangular network with next-nearest-neighbour connections can be manufactured with the same method^72^. The biocompatible materials for ANN fabrication are polydimethylsiloxane (PDMS) parylene^73^ and hydrogels^74^. With structures like through-holes, neuron cells can be immobilised and connected and using micro tunnels, which can be encoded into a layer of PDMS or hydrogel for neurite outgrowth nanodiamond (ND) layering^75^ has also been demonstrated to be an excellent growth substrate for functional neuronal networks with the aid of photolithography methods.

After the fabrication of a scaffold, the ANN with desired topology can be achieved by cell placement, which is usually the combination of cell delivery and localisation. Cell placement is usually feasible using glass micro-pipettes^76^, microfluidics, laser guidance^77^ and micromanipulation systems^78^ for pick-and-place positioning.

### Fabrication of direct ANNs in two dimensions

The current methods for building direct ANNs based on biological neurons are largely limited to two-dimensional (2D) substrates. Such 2D neural cultures cannot achieve the 3D spatial extensions of axons and dendrites. However, BNNs in the human brain possess extraordinary connectivity and complexity from the millimetre-scale down to a scale of several nanometres in 3D, which include the microscale of single neurons and synapses (nm–μm, synapse-by-synapse), the mesoscale of neuronal populations and their interconnecting circuitry (region-by-region), and the macroscale of anatomically distinct brain regions and pathways. Consequently, the development of accurate ANNs that mimic the brain remains a significant obstacle to our understanding of the functioning of the brain at different levels.

Recent developments in using 3D additive printing technique demonstrated it great capability potential in building large-scale 3D ANNs (Fig. 6d–f). Hydrogel-made 3D brain-like structures consisting of primary neurons have been created with a peptide-modified biopolymer^79^. This technique has been used for the creation of intricate functionalized 3D brain-like modular formed from cortical tissue, maintained alive for months in vitro. 3D architectures that compartmentalising biological tissues have also been achieved by 3D additive printing using silk-collagen protein scaffolds^80^ and hydrogels^81^, which have then been used to build 3D brain-like tissue seeded with primary cortical neurons.

One clear disadvantage of these 3D ANNs is that the connections between neurons are randomly patterned. The fabrication resolution of traditional 3D additive printing limits its capability of creating 3D ANNs with micrometre- or nanometre scale resolution.

### Towards building direct ANNs based on biological neurons with nanoscale resolution in three dimensions

One potential solution towards the building of 3D and nanometre scale ANNs lies in the recent development of 3D DLW. 3D DLW, such as single-beam two-photon DLW and two-beam SPIN^23,82^, has been widely studied and utilised to produce 3D nanophotonic structures^82^, holograms^46^, microfluidics^83^, biomedical implants^84,85^, 3D scaffolds for cell cultures and tissue engineering^86,87^ and biomimetic neuron structures^88–91^.

Owing to an intrinsic ability to produce 3D structures with a wide range of photosensitive materials, single-beam two-photon DLW has been used to fabricate scaffolds for ANNs with biological cells to study the growth of neurons and guidance^92^, as shown in Fig. 6g–i. Low-profile barrier structures have been successfully fabricated using bovine serum albumin (BSA) and laminin to guide the interactions of brain cortical neurons and neuroblastoma–glioma hybrid cells (NG108-15) in neuron cell culture^93,94^. Using hyaluronic acid hydrogels, guidance pathways of biotinylated BSA functionalized with IKVAV peptides for rat dorsal root ganglion cells and rat hippocampal neural progenitor cells have also been created^95^. 2PP have also been used to create scaffold using synthetic biodegradable polymers. 3D structures have been fabricated using polylactide-based photopolymer with the shape of linear guidewires, with which the directed growth behaviour of NG108-15 and PC12 neuroblastoma cells have been studied^96^.

### Future perspectives

Intense ongoing research activity is focused on the use of two-photon DLW in the fabrication of 3D scaffolds for neuron growth and neuron tissue regeneration. Two-photon DLW is the only technique able to provide the 3D fabrication capability and high spatial resolution that are necessary to create a scaffold mimicking the structural features of BNNs. This technique may offer the means to produce scaffolds that are bioactive at micrometre scale and even at nanometre scale.

Modification of the physical and chemical characteristics of 3D nano/microstructures with submicron resolution will act as a crucial key in the study of BNNs. Because of the unique electrical and optical properties assembled on these platforms, they also hold the potential to augment brain functions. Creation of 3D nano/microstructures-based biosensors is faced with many challenges, but the aim is to achieve single electrical connections in brain neuron circuits and neural networks of interest. Eventually, nano/microstructures manifest the interactive platform between nanotechnology and neuroscience, making them promising medium in neuron technology for diagnosing and treatment of brain diseases in neurology.

## Signal detection of direct ANNs enabled by nanophotonics

Unlike indirect ANNs based on electronics and photonics with input and output connections for signal detections, the spiking of biological neurons are the signals of direct ANNs. The detection of such weak electric signals at the nanoscale is of critical importance to studying neural activities. Novel devices that are ultrasensitive to weak electric fields, such as micro-electrode arrays and patch clamps, have been introduced to study neural activities. However, these devices have a low spatial resolution and are invasive, which might cause damage to the biological neurons. Nanophotonics, especially nanosensing enabled by nitrogen-vacancy (NV^−^) centres in bulk diamonds and NDs, has opened the possibility of the far-field detection of neural activities with nanoscale resolution.

### Sensing of the action potential

The electrical potential associated with the passage of a spiking impulse along neurons is called action potential (AP). APs play a central role in the communication between individual neurons. The signals between neurons propagate along axons via APs. Different techniques can be implemented for the measurement of APs. Electrophysiology is a recording method with a patch-clamping configuration that remains the gold standard for the measurement of individual APs. The technique has an excellent temporal resolution and good signal-to-noise ratio, but the spatial resolution is limited to ~ 10 µm.

Nanophotonic techniques to measure APs offer many advantages, but they typically require high power, which can cause photodamage to neurons. In addition, voltage-sensitive fluorescent proteins must be genetically expressed, which may alter the neuronal functions. Owing to their optical and magnetic properties, NV^−^ centres can be applied to study neural networks and the firing of neural cells to study brain activity^97^.

NV^−^ centres have attracted significant attention in the recent years owing to their outstanding optical and magnetic properties^98,99^. The ground state of an NV^−^ centre is a triplet state with six unpaired electron spins, each one with an associated magnetic moment; the spin sublevels are *m*_s_ = ±1,0^100^. The energy sublevels *m*_s_ = ±1 in the ground state can be perturbed with an external magnetic field. This paramagnetism of the ground state can be mathematically described by the spin Hamiltonian of the system^100^:

3 $$H = D_{gs}S_z^2 + E_{gs}\left( {S_x^2 - S_y^2} \right) + g_{gs}\mu \overrightarrow B \cdot \overrightarrow S$$

where *D*_*gs*_ is the ground state zero-field splitting, $\vec S$ is the spin operator vector, *E*_*gs*_ is the ground state strain-induced splitting coefficient, *g*_*gs*_ the ground state *g*-factor and *µ* is the Bohr magneton^101^. In brief, the first term corresponds to the energy gap between the two sublevels in the ground state, the second term represents the energy shift owing to the strain from the lattice, and the last term is the Zeeman effect^102,103^.

When NDs are stimulated by a microwave (mw) field, it is possible to redistribute the population of ground state electrons in NV^−^ centres. In particular, by switching on an mw field at the zero-field splitting frequency, *D* = 2.87 GHz, there is a redistribution of the electrons from the most populated sublevel, *m*_*s*_ = 0, to the less-populated sublevel, *m*_*s*_ = ±1.

Under an excitation beam at wavelength 532 nm, an NV^−^ centre is polarised in its ground state *m*_s_ = 0. The application of an mw field at the zero-field splitting frequency causes an increase in the population of the *m*_*s*_ = ±1 spin sublevels. This leads to an overall decrease in the fluorescence because of the non-radiative decay via the intermediate metastable state. The observed photoluminescence decrease is called the optically detected magnetic resonance (ODMR) signal. It is also possible to apply a magnetic field along the *z* axis that degenerates the energy sublevels *m*_*s*_ = ±1.

In case of the weak-magnetic field regime, the magnetic field is calculated with the following^104^:

4 $$B_{NV} = \frac{{\omega _2 - \omega _1}}{{2g_{gs}\mu }}$$

Under this circumstance, the recorded ODMR signal shows two dips owing to the redistribution of the electrons in the sublevels *m*_*s*_ = −1 and *m*_*s*_ = +1. The separation between the recorded ODMR dips increases with the strength of the applied magnetic field.

In fact, APs are time-varying electrical fields, which lead to time-varying magnetic fields. The main principle behind sensing APs with NV^−^ centres is the detection of time-varying magnetic fields generated by APs via ODMR signals. The measurement of the AP via magnetic field sensing confers important advantages: it is non-invasive, label-free and able to detect neuronal activity through intervening tissue and whole organisms.

In the first work of applying NV^−^ centres to measure the axon transmembrane potential, the magnetic field generated by a single axon potential is modelled, magnetic field generated by a single axon potential is modelled and the magnetic field has been generated by a microwire on the surface of a diamond substrate, which simulates the AP of a morphologically reconstructed hippocampal CA1 pyramidal neuron. The detection system is composed of a commercial grade single crystal ultra-pure diamond substrate with a layer NV^−^ defect centres with a standoff of 100 nm. The detection system is able to image planar neuron dynamics non-invasively with temporal resolution at millisecond and spatial resolution at micron spatial resolution (10 µmTHz^−1/2^) within wide-field view^105^. NV^−^ centres in bulk diamonds have been implemented to measure the APs of a giant axon in an invertebrate, *Myxicola infundibulum*^97^.

**Fig. 7:**
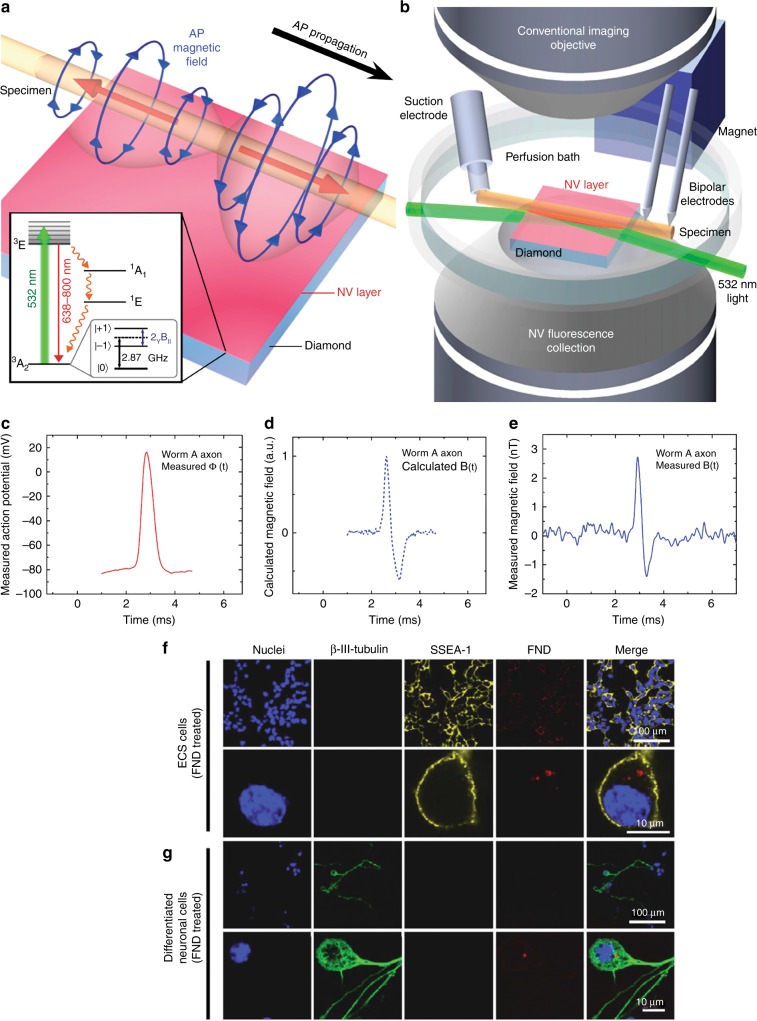
Experimental overview of the measurement of action potential (AP) with NV centres^97^ and detection and location of nanodiamonds (NDs) in ECS cells^107^. **a** A schematic of the bipolar azimuthal magnetic field associated with a giant axon. Inset: the energy-level diagram of an NV^−^ centre. **b** A schematic of the microscope implemented for the magnetic measurements. **c** Measured AP voltage with the electrophysiology measurement. **d** Calculated magnetic field. **e** Measured magnetic field with NV^−^ centres. **f** ECS cells treated with NDs. In red is the fluorescence intensity of NDs excited with light at a wavelength of 580 nm. The emission is collected in the wavelength range of 600–700 nm. In blue is the nucleus of the cell, and in yellow, the SSEA-1. **g** Differentiated neural cells. Fluorescent NDs are observed in the cytoplasm of undifferentiated ECS and differentiated cells

The bipolar azimuthal magnetic field associated with an AP is depicted in Fig. 7a, in the inset is the energy level of the NV^−^ centres. A light beam at a wavelength of 532 nm is applied to the sensing NV^−^ centre layer through the diamond at a sufficiently shallow angle so that the light reflects off the top diamond surface and therefore does not irradiate the living sample (Fig. 7b)^97^. The magnetic field from the AP measured via electrophysiology can be mathematically expressed as^97^:

5 $$\phi _{in}(t):B(t) = {\int} {sd\phi _{in}dt}$$

The measured AP voltage and the calculated magnetic field are illustrated in Fig. 7c–e. The measurements with NV^−^ centres are consistent with the prediction^97^.

With NV^−^ centres, it is also possible to measure the AP propagation direction at a single point^97^. The results lay the groundwork for the real time, non-invasive 3D magnetic mapping of functional neuronal networks, ultimately with a circuit-scale (~ 1 cm) field-of-view.

### Labelling and tracking of neuronal differentiation with NDs

An understanding of biological processes involves the tracking of several cells to study their differentiation and development. Owing to their photostability, their robustness against photobleaching and their biocompatibility, NDs are ideal candidates for long-term cell tracking^106^. A study has been reported on the tracking of neuron cells derived from a model of embryonal carcinoma stem cells with fluorescent NDs^107^. The NDs are detected with a confocal microscope (Fig. 7f). No effect on the morphological development of the cells is recorded nor do the NDs induce apoptosis during the neuronal differentiation (Fig. 7g). Therefore, the implementation of fluorescent NDs to track neural cell development could provide potential therapeutics for neural diseases^107^.

### Future perspectives

Studies on the detection of APs by measuring the ODMR signal with NV^−^ centres in bulk diamonds have been reported^97^. There are several technical challenges with this technique. First, the magnetic field sensitivity needs to be improved to enable AP measurement from individual mammalian neurons^97^. Second, single-point detection needs to be developed for the 2D and 3D mapping of APs in neuronal networks. Third, the high-resolution imaging of AP magnetic fields is also required.

NDs can provide a new solution to the three challenges listed above. NDs has a wide range of applications in the life sciences in the life sciences compared with bulk diamonds^108^. Currently, NV^−^ centres in NDs have been applied to study the formation and patterning of neural networks and to label and track the neuron cells^75,107^. First, a higher density of NV^−^ centres can yield a sensitivity improvement. NDs provide a substantially higher density of NV^−^ centres than does bulk diamond. Some types of NDs contain ~ 1000 NV^−^ centres in one ND with a diameter of 5 nm. Second, NDs enable 3D magnetic imaging. The magnetic imaging based on diamond layers can only achieve 2D imaging because the neuron networks can only be fixed on the surface of the diamond layers. However, NDs can be labelled everywhere inside the neuron networks, which makes it possible to obtain 3D magnetic images. Third, a higher spatial resolution can be achieved with NDs. Smaller NDs in biomedical imaging are highly required, and the blinking phenomenon is more prevalent when reducing the size of a ND is reduced^109–111^. Super-resolution imaging based on blinking fluorescence would allow the nanometric reconstruction of synapse connections by labelling the neuron cells with 5 nm oxide NDs^109,111^.

The employment of the ODMR signal to interpret APs via magnetic field measurements could lay new groundwork for the development of nanoscale biomarkers for both the super-resolution optical imaging and magnetic sensing of ANNs.

## Conclusions

In the present review, we have surveyed the recent advancements in nanophotonic technology for indirect and direct ANNs. Holography and integrated photonic circuits have shown potential for achieving indirect ANNs. To date, the integration density of functional devices in ANNs based on photonic circuits remains limited. Three-dimensional photonic integration enabled by DLW could be a solution to the development of indirect ANNs with a high bandwidth and low-power consumption. DLW can be used for the direct fabrication of ANNs in three dimensions. In addition, photonic sensing techniques can be used to study the neural activities in direct ANNs based on biological neurons. Further developments in nanofabrication and super-resolution optical sensing techniques are essential to achieve fabrication and detection capabilities at the scale of the ANN nanofeatures.
